# Toward fMRI-based hyperscanning in the study of the cocktail-party effect

**DOI:** 10.3389/fnins.2026.1806385

**Published:** 2026-03-25

**Authors:** Qiang Li

**Affiliations:** College of Education Science, Guizhou Education University, Guiyang, China

**Keywords:** EEG, fMRI, fNIRS, hyperscanning, the cocktail-party effect

## Introduction

In everyday life, humans are constantly immersed in acoustically complex environments, yet they can effortlessly focus on a single attended speech stream while suppressing other competing unattended streams. This remarkable ability is known as the cocktail-party effect (Ahmed et al., 2023). Understanding how the central nervous system selectively extracts relevant auditory information while suppressing competing inputs, and identifying the neural mechanisms underlying this process, have long been central topics in auditory and cognitive neuroscience (Bednar and Lalor, 2020).

Recent studies have revealed that the cocktail-party effect is not solely a property of individual brains, but also involves inter-brain neural synchronization between communicating individuals **(**Holtze et al., 2022**)**. Compared with traditional brain-sound neural resonance approaches (i.e., brain-to-stimulus coupling), inter-brain synchronization directly examines neural alignment between interacting brains (Rosenkranz et al., 2021), bypassing the acoustic signal as an intermediate representation. This perspective offers a more direct window into the neural mechanisms underlying selective listening in natural communication. Clarifying how attended and unattended streams differentially shape inter-brain coupling is therefore crucial not only for understanding the neural basis of the cocktail-party effect, but also for informing the development of brain-brain interfaces under realistic multi-talker conditions.

## Limitations of existing approaches

To date, most research on the neural mechanisms of the cocktail-party effect has focused on neural tracking of speech signals, using EEG, MEG, or fNIRS (Ahmed et al., 2023; Keshavarzi and Varano, 2021; Mesgarani and Chang, 2012; O'Sullivan et al., 2019). These studies have provided compelling evidence that attended speech is preferentially represented at higher levels of the auditory hierarchy, while unattended speech is largely confined to early auditory cortex.

However, speech signals themselves are the result of multiple stages of neural processing and complex articulatory transformations. As such, brain-sound coupling inevitably reflects a mixture of sensory encoding, motor production, and environmental distortion. In contrast, directly measuring neural signals from interacting brains allows researchers to examine the neural dynamics of communication at their source. Inter-brain synchronization therefore offers a more direct and theoretically grounded approach for probing the neural basis of selective listening in cocktail-party scenarios.

Despite its promise, inter-brain research on the cocktail-party effect remains underdeveloped. Most existing studies rely on EEG or fNIRS (Dai et al., 2018; Holtze et al., 2022; Kuhlen et al., 2012; Li J. et al., 2023; Li et al., 2021; Li Z. et al., 2023; Rosenkranz et al., 2021), which, although sensitive to temporal dynamics, suffer from limited spatial resolution and are largely insensitive to deep-brain (subcortical) activity. Consequently, these studies are unable to precisely localize where inter-brain synchronization emerges, particularly within deeper cortical and subcortical regions, nor can they adequately distinguish the neural mechanisms supporting attended vs. unattended streams.

## Advantages of fMRI-based hyperscanning

fMRI-based hyperscanning has emerged in recent years as a powerful tool for studying neural coupling during social communication **(**Hausfeld et al., 2024; Liu et al., 2020; Speer et al., 2024; Stephens et al., 2010; Xie et al., 2020**)**. Compared with EEG, MEG, and fNIRS, fMRI offers superior spatial resolution and whole-brain coverage that includes both cortical and subcortical structures, while avoiding the invasiveness and limited applicability of electrocorticography (ECoG).

The high spatial precision of fMRI hyperscanning is particularly advantageous for research on the neural mechanisms of the cocktail-party effect. It enables accurate localization of neural activity and inter-brain synchronization across distributed cortical and subcortical systems involved in speech processing and attentional control. This opens the door to disentangling the neural substrates associated with attended vs. unattended speech at multiple hierarchical levels, a distinction that remains difficult to achieve with existing noninvasive techniques. Importantly, compared with EEG- or fNIRS-based hyperscanning, which offers only coarse and spatially limited measures of inter-brain coupling, fMRI hyperscanning provides anatomically precise mapping of cross-brain synchrony. This allows researchers to identify the specific networks involved and to advance from descriptive observations toward mechanistic explanations of how selective listening shapes communication-related circuitry.

## Methodological challenges and practical solutions

Applying fMRI hyperscanning to cocktail-party effect paradigms is not without challenges. First, MRI scanners generate substantial acoustic noise, imposing stringent requirements on real-time speech denoising. Second, the scanner environment precludes face-to-face interaction, potentially reducing ecological validity. Third, true hyperscanning beyond dyads would require the simultaneous operation of three or more MRI scanners, which poses substantial logistical, financial, and technical challenges. Fourth, compared with indirect neuroimaging techniques such as EEG and fNIRS, fMRI has relatively limited temporal resolution, which may constrain the investigation of rapid, dynamic neural processes underlying spoken language communication.

These challenges, however, are not insurmountable. A staged approach that incrementally balances technical feasibility with scientific ambition may offer a pragmatic pathway. In the first stage, pseudo-hyperscanning paradigms can be employed to lower initial technical barriers and achieve partial objectives such as establishing robust inter-brain coupling metrics, validating speech denoising pipelines, and refining stimulus design under controlled conditions. In such designs, neural signals are first recorded from multiple speakers during speech production, and their denoised speech signals are then combined offline to construct cocktail-party stimuli subsequently presented to listeners during fMRI scanning. This approach preserves key advantages of hyperscanning—namely inter-brain coupling analysis—while dramatically reducing immediate technical demands and scanner requirements.

In the second stage, fully real hyperscanning research using simultaneous fMRI of multiple participants should be undertaken to address real-time cocktail-party language communication. In this phase, pairs or triplets of interacting participants are scanned simultaneously in different MR scanners with synchronized acquisition. Each speaker's speech signal should undergo real-time denoising prior to mixing, independent of the experimental manipulation. The two denoised streams can then be adaptively combined to construct a cocktail-party acoustic scene, with mixing parameters experimentally controlled (e.g., via spatial or speaker-identity cues) before real-time presentation to the listener inside the scanner. To enhance ecological validity, a video interface displaying the communication partners can be incorporated so that participants engage in more face-to-face–like interaction despite the physical constraints of the scanner environment.

In parallel with experimental implementation, the analytical framework for inter-brain data should be explicitly specified. Several established and complementary approaches can be employed for fMRI hyperscanning data analysis. First, inter-brain functional coupling can be quantified using Pearson correlation, which has been extensively validated in functional connectivity research and provides a straightforward measure of temporal synchrony between homologous or functionally defined regions across participants (Biswal et al., 1995). Despite its simplicity, correlation-based inter-subject coupling has proven robust in naturalistic paradigms, including narrative communication (Stephens et al., 2010). Second, multivariate linear regression models offer important advantages by enabling the explicit modeling and removal of confounding factors, such as head motion parameters, physiological noise regressors, scanner drift, and task-related covariates within a general linear model (GLM) framework (Satterthwaite et al., 2013). Such approaches improve the specificity of inter-brain coupling estimates by reducing shared artifactual variance, which is particularly critical in hyperscanning contexts where motion and acoustic artifacts may be correlated across participants. Third, wavelet coherence analysis provides a powerful time–frequency framework for assessing inter-brain coupling across multiple temporal scales. Unlike static correlation measures, wavelet coherence can characterize non-stationary and frequency-specific synchronization patterns, making it especially suitable for investigating dynamic social interaction and speech-related rhythms (Chang and Glover, 2010). Given that conversational speech contains hierarchical temporal structure (e.g., syllabic and phrasal rhythms), time–frequency approaches may reveal frequency-dependent inter-brain alignment that is not captured by stationary metrics.

To further validate inter-brain coupling measures, stimulus-driven benchmarks can be incorporated. Specifically, the speech amplitude envelope of each speaker can be convolved with a canonical hemodynamic response function (HRF) and correlated with the listener's BOLD signal. Such speech–brain coupling analyses have been widely used to quantify neural entrainment to continuous speech (Lerner et al., 2011). Convergence between envelope-based stimulus–brain coupling and inter-brain coupling metrics would provide an important cross-validation of the analytical framework, ensuring that observed interpersonal neural alignment reflects meaningful speech-driven processes rather than shared noise or scanner-related artifacts.

A key limitation of conventional fMRI is its relatively low temporal resolution compared with techniques like EEG or MEG, which may constrain the characterization of rapid dynamic neural processes. However, recent advances in multiband (simultaneous multi-slice, SMS) have substantially improved temporal sampling. For example, multiband SMS protocols have enabled whole-brain coverage with repetition times (TRs) reduced from the typical 2–3 s to sub-second ranges such as ~0.72 s in large population projects like the Human Connectome Project (Wall, 2023), thereby increasing the effective sampling rate of BOLD signals. More sophisticated hybrid methods combining multiband encoding with advanced readout strategies (e.g., echo-volumar encoding) have been developed that sustainably support even shorter TRs (e.g., 118–650 ms) without sacrificing whole-brain coverage and with maintained temporal SNR, enabling the sensitive mapping of neural processes at higher frequency bands (e.g., above 0.3 Hz) (Posse et al., 2025). At the extreme, proof-of-principle work combining multiband acceleration with innovative reshuffling strategies has demonstrated effective BOLD sampling rates of ~75 ms, which would, according to Nyquist's sampling theorem, support neural signal components up to ~6–7 Hz in principle (Schmidt et al., 2023). Importantly, these accelerated sampling regimes also make it feasible—at least from a sampling-adequacy perspective—to probe faster neural responses aligned with speech temporal structure, including syllabic (~1.5 Hz) and phrasal (~3 Hz) rhythmic components (Meng et al., 2021). Such improvements in temporal resolution bring fMRI closer to the timescales of many aspects of speech and language exchange, making real-time hyperscanning more feasible.

Collectively, these methodological advances establish a scalable framework for investigating the neural mechanisms underlying the cocktail-party effect through fMRI hyperscanning, bridging controlled experimental models and real-time multi-speaker social interaction.

## Beyond a unitary cocktail-party effect

Finally, it is worth noting that the cocktail-party effect is not a single, homogeneous phenomenon. Selective listening can rely on multiple cues, including voice identity, timbre, semantics, and spatial location (Liu et al., 2024). Yet many studies treat these diverse mechanisms as manifestations of a single effect. The spatial resolution of fMRI hyperscanning makes it possible to systematically dissociate the neural mechanisms supporting different cue-based forms of selective listening, thereby refining theoretical understanding of the cocktail-party effect.

## Conclusion

fMRI-based hyperscanning offers a powerful and timely approach for advancing research on the neural mechanisms underlying the cocktail-party effect. By leveraging its high spatial resolution and whole-brain coverage, this method enables systematic dissociation of the neural processes supporting different cue-based forms of selective listening, thereby overcoming key conceptual and methodological limitations of prior approaches. Integrating fMRI hyperscanning into research on the cocktail-party effect thus provides a more precise mechanistic framework for understanding how brains dynamically coordinate during complex communicative environments.
